# Hybrid Macro-Porous Titanium Ornamented by Degradable 3D Gel/nHA Micro-Scaffolds for Bone Tissue Regeneration

**DOI:** 10.3390/ijms17040575

**Published:** 2016-04-15

**Authors:** Bo Yin, Pei Ma, Jun Chen, Hai Wang, Gui Wu, Bo Li, Qiang Li, Zhifeng Huang, Guixing Qiu, Zhihong Wu

**Affiliations:** 1 Department of Orthopaedic Surgery, Peking Union Medical College Hospital, Peking Union Medical College and Chinese Academy of Medical Sciences, No. 1 Shuaifuyuan, Beijing 100730, China; yinbo1716@163.com (B.Y.); dr_mapei@163.com (P.M.); xixiangchenjun@163.com (J.C.); wanghai19801117@163.com (H.W.); wugui_1985@sina.com (G.W.); 329305278@163.com (B.L.); lq46039251678@126.com (Q.L.); hzfpumch@hotmail.com (Z.H.); qgx1718@126.com (G.Q.); 2 Institute of Materia Medica, Chinese Academy of Medical Sciences and Peking Union Medical College, Beijing 100050, China; 3 Central Laboratory, Peking Union Medical College Hospital, Peking Union Medical College and Chinese Academy of Medical Sciences, No. 1 Shuaifuyuan, Beijing 100730, China; 4 Beijing Key Laboratory for Genetic Research of Bone and Joint Disease, No. 1 Shuaifuyuan, Beijing 100730, China

**Keywords:** porous titanium, Gel/nHA, 3D micro-scaffolds, hybrid scaffold, osteogenesis

## Abstract

Porous titanium is a kind of promising material for bone substitution, while its bio-inert property results in demand of modifications to improve the osteointegration capacity. In this study, gelatin (Gel) and nano-hydroxyapatite (nHA) were used to construct 3D micro-scaffolds in the pores of porous titanium in the ratios of Gel:nHA = 1:0, Gel:nHA = 1:1, and Gel:nHA = 1:3, respectively. Cell attachment and proliferation, and gene and protein expression levels of osteogenic markers were evaluated in MC3T3-E1 cells, followed by bone regeneration assessment in a rabbit radius defect model. All hybrid scaffolds with different composition ratio were found to have significant promotional effects in cell adhesion, proliferation and differentiation, in which the group with Gel:nHA = 1:1 showed the best performance *in vitro*, as well as the most bone regeneration volume *in vivo*. This 3D micro-scaffolds modification may be an innovative method for porous titanium ornamentation and shows potential application values in clinic.

## 1. Introduction

Critical-size bone defects caused by trauma or tumors cannot be repaired by the body itself, making bone graft the second most common transplant in the clinical setting [1,2]. Although autologous bone from iliac crest and fibula has been considered as the gold standard for bone grafting [3], allogeneic bone has also been widely used [4]. However, several disadvantages, such as relatively high rate of donor-site trauma, infection, limited bone mass, disease transmission and immunogenicity cannot be neglected [5,6]. Tissue-engineered bone substitutes, including natural and synthetic materials, such as collagen, demineralized bone matrix (DBM), polylactic acid (PLA), polylactic-co-glycolic acid (PLGA), hydroxyapatite (HA) and tricalcium-phosphates (TCP), are therefore considered to be the most promising alternatives [7]. However, several insurmountable disadvantages, such as lack of strength [8,9,10], a mismatch between materials degradation and new bone growth [11,12,13,14,15,16] still existed, despite good biocompatibility and degradation performance.

Titanium alloy is a kind of promising material, which has been used in clinical application for its safety and excellent mechanical properties [17,18], above that, its excellent chemical inertness, corrosion resistance, re-passivation ability and biocompatibility are thought to result from chemical stability [19]. Using a 3D printing technique, the porosity of titanium scaffold can achieve up to 80% which mimics the porous structure of bone, insures mechanical support, and provides spaces for bone growth and nutrient supply [20,21]. In addition, this kind of porous titanium scaffold can reduce the stress shielding effects [22]. However, titanium is a kind of bio-inert material, and certain modifications should be required to endow it with biomechanical stability, osteoconduction and osteoinduction as well as provide it a favorable environment for neovascularization and bone regeneration [7]. Various modifications on porous titanium, such as chemical treatment, plasma coating and bioactive layer, have been reported. Unfortunately, most of these modifications focused on the inner surface of the porous structure in two-dimensional levels [4,23,24,25,26,27]. Previous studies suggested proper pore size of 100–300 μm [28,29,30,31]. In recent years, the pore size of porous titanium used in clinical studies or research reaches 500–1500 μm. However, few studies have been performed to investigate the osteogenic effect of large aperture porous titanium. In the present study, by using a new 3D modification method, we constructed micro-scaffolds in the pores of macro-porous titanium. This hybrid scaffold with appropriate 3D micro-structure was proved to be able to promote new bone growth.

Collagen and hydroxyapatite are recognized as biological material with good osteoconduction and estoinduction capacity [32,33]. Collagen is the main component of bone matrix. Hydroxyapatite (HA) is a synthetic biomaterial, which is commonly used in bone tissue repair and augmentation for its biocompatibility and surface active properties; nano-sized HA, in particular, exhibited enhanced resorbability and greater bioactivity than micron-sized HA (mHA) [26]. In this study, we used three proportion ratios of collagen-derived gelatin (Gel) and nano-hydroxyapatite (nHA) (Gel:nHA = 1:0, Gel:nHA = 1:1, and Gel:nHA = 1:3) to construct the 3D micro-scaffolds with suitable pore size. Then, the morphological characteristics, biocompatibility and osteogenesis ability of hybrid scaffolds were further evaluated *in vitro* and *in vivo*, followed by comparison of these measurements with non-modified porous titanium.

## 2. Results

### 2.1. Implant Characterization

**Figure 1:**
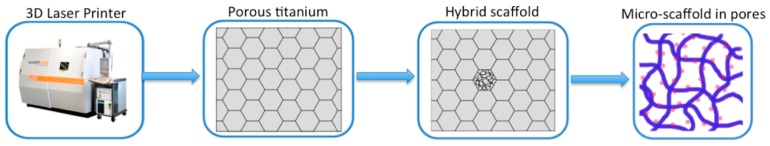
Schematic of the fabrication process of hybrid scaffolds.

**Figure 2:**
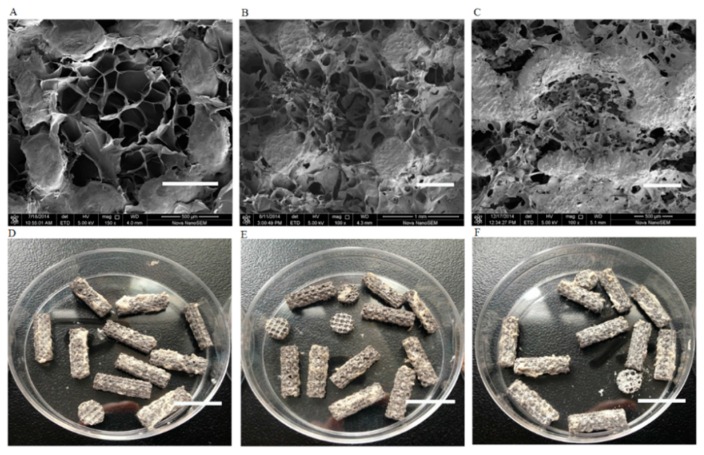
Surface characterization and gross view of hybrid scaffolds by scanning electron micrograph (SEM). Gel:nHA = 1:0 group (**A**,**D**); Gel:nHA = 1:1 group (**B**,**E**); Gel:nHA = 1:3 group (**C**,**F**). Scale bar: 500 μm (**A**–**C**), 15 mm (**D**–**F**).

As shown in Figure 1, porous titanium implants were produced with a porosity of 80.7% ± 4.6%, a strut size of 352 ± 46 μm, a compressive strength of 77.4 ± 3.6 MPa, and a modulus of elasticity of 3.4 ± 0.8 GPa, which were close to the theoretical values and similar to those of human trabecular bone. Macroscopic inspection and scanning electron micrograph (SEM) analysis verified that pores of the porous titanium were completely filled with Gel/nHA micro-scaffolds (Figure 2). SEM showed significant differences among three groups. Group 1 with Gel:nHA = 1:1 had a multiple-hierarchical structure with the average pore size of 156 ± 86 μm, which was closest to the normal human bone (Figure 2B,E); group 2 with Gel:nHA = 1:0 showed uniform but rather compact pores with the average size of 67 ± 32 μm (Figure 2A,D); and the last group with Gel:nHA = 1:3 showed lots of collapsed areas, resulting in more invalid space (Figure 2C,F).

### 2.2. Cell Attachment and Proliferation

**Figure 3:**
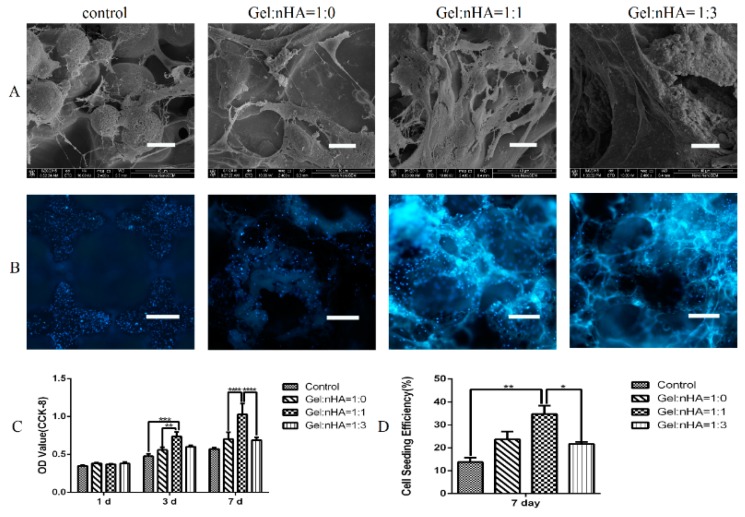
Cell attachment and proliferation on hybrid scaffolds. (**A**) morphology of cells attached on different scaffolds; (**B**) cell seeding efficiency based on DAPI staining of cells attached on different scaffolds (100×); (**C**) cell proliferation measured based on optical density (OD )value by CCK-8 assay; (**D**) cell seeding efficiency calculated by ImageJ software (NIH, Bethesda, MD, USA). Scale bar: 20 μm (**A**), 500 μm (**B**). * *p* < 0.05, ** *p* < 0.01, *** *p* < 0.001, **** *p* < 0.0001.

After a seven day culture, cells were observed with different distributions by SEM (Figure 3A). Especially, in Gel:nHA = 1:1 group, cells presented elongated and plump morphology with abundant filamentous and noticeable filopodias, while in the other three groups, sparsely distributed cells were observed with atrophied and less pseudopodium.

During a seven day culture, steadily increased cell proliferation was observed by using a CCK-8 assay, and the group with Gel:nHA = 1:1 showed significant cell proliferation at day 3 and day 7. To qualitatively evaluate live cells on the composite scaffolds, MC3T3-E1 cells were stained with 4’,6-diamidino-2-phenylindole (DAPI) (Figure 3B,D). As expected from the CCK-8 result (Figure 3C), Gel:nHA = 1:1 group showed a significantly higher cell seeding efficiency (up to 25%–30%) after a seven day culture than that in other groups. All of these suggested that the ratio of Gel:nHA = 1:1 may provide a more suitable microenvironment for cell adhesion and proliferation.

### 2.3. Cell Differentiation

**Figure 4:**
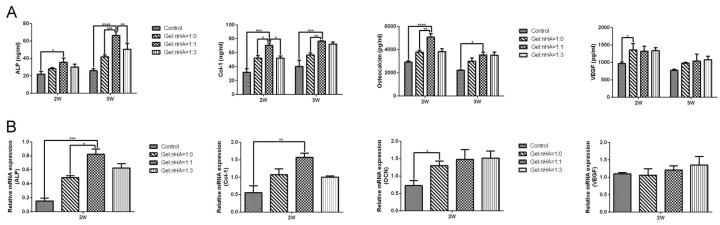
Osteogenesis differentiations of attached cells was assessed by ELISA (**A**) and PCR (**B**) using osteogenic (ALP, Col-1, OCN) and angiogenic (VEGF) markers at 2 weeks and 3 weeks. * *p* < 0.05, ** *p* < 0.01, *** *p* < 0.001, **** *p* < 0.0001.

ELISA test performed at day 14 and day 21 showed significantly upregulated expression of alkaline phosphatase (ALP), collagen-1 (Col-1), and osteocalcin (OCN) (*p* < 0.05) in Gel:nHA = 1:1 group; no difference was found for vascular endothelial growth factor (VEGF) among four groups (Figure 4A). Correspondingly, (Figure 4B) gene expressions of ALP and Col-1 were significantly higher in Gel:nHA = 1:1 group (Figure 4A,B), whereas OCN and VEGF showed no significant difference.

### 2.4. Micro-CT Evaluation

**Figure 5:**
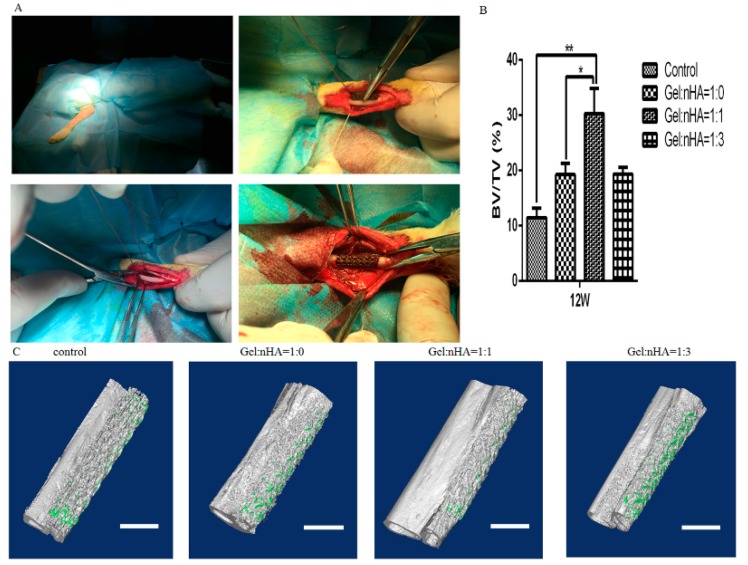
Surgical procedure and bone formation effects of different scaffolds. (**A**) surgical procedure; (**B**) BV/TV calculated by the CTAn software (Bruker Co. Kartuizersweg, Kontich, Belgium); (**C**) the volume of regenerated bone reconstruction based on *ex vivo* micro-CT scans at 12 weeks. Scale bar: 4 mm. * *p* < 0.05, ** *p* < 0.01.

In total, 24 rabbits were included in the study with no other surgical complications and no dislocation. Bone ingrowth started from the host bone bed towards the implant treatment with Gel/nHA 3D micro-scaffolds resulting in higher percent bone volume (BV/TV) calculated by CTAnalyser than that in the empty controls after 12 weeks (Figure 5), and BV/TV reached the highest percent in Gel:nHA = 1:1 group (30.3% ± 1.6%), which could be verified on micro-CT reconstruction images.

### 2.5. Histological Evaluation

**Figure 6:**
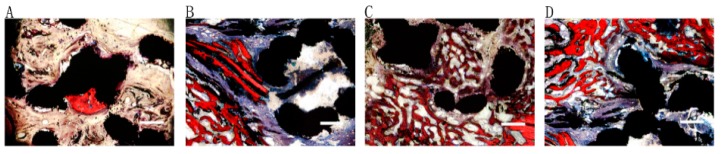
Histological analysis of hybrid implants osteointegration ability in control group (**A**) Gel:nHA = 1:0 group; (**B**) Gel:nHA = 1:1 group; (**C**) Gel:nHA = 1:3 group; (**D**) by Van Gieson staining at 12 weeks. (red indicated new bone formation, blue represented fibrous tissue and black showed the metal). Scale bar: 500 μm.

As shown in Figure 6, no distinct difference in terms of bone morphology, bone-titanium bonding or vascularization among groups was observed. The pores of porous titanium scaffold were filled with newly formed bone, and new bone areas in Gel:nHA = 1:1 (Figure 6C) hybrid scaffolds were significantly bigger than that in the Gel:nHA = 1:0 (Figure 6B) and Gel:nHA = 1:3 (Figure 6D) groups. Rare bone regeneration could be seen in the control group.

## 3. Discussion

In this study, 3D Gel/nHA micro-scaffolds were used as a modification method to improve bone regeneration ability of porous titanium. *In vivo* experiments showed that the hybrid scaffolds could enhance bone regeneration in critical-sized radius bone defects in rabbits. Upregulated expressions of osteogenic genes and proteins were observed *in vitro* after hybrid scaffold modification. The suitable pore size and favorable environment provided by this hybrid scaffold may contribute to bone regeneration.

Titanium alloy implants have been highly praised and widely used for many years in the clinic. With the advent of 3D printing technology, titanium alloy can be prepared with personalized design, high porosity and appropriate elastic modulus to meet clinical needs [34,35]. The more than 80% porosity (over 80% spaces) can be used to accommodate new bone. In addition, as a kind of non-absorbable material, titanium can provide durable mechanical support. Surgeons need not worry about the mismatch between material degradation and the new bone growth rate, thus avoiding serious consequences [36,37].

Studies have revealed that several properties of scaffolds may affect bone regeneration, such as mechanical properties, surface character, bioactive factors and internal architecture [7]. In our study, porous titanium implant was produced with the compressive strength of 77.4 ± 3.6 MPa, the elastic modulus of 3.4 ± 0.8 GPa and the porosity of 80.7% ± 4.6%, which are similar to the mechanical properties of natural bones [38,39]. Previous studies reported that neither small nor large pore size was conducive for cell growth, and pore size or crosslinking density also have an impact on biomolecule diffusion [40], and the recommended pore size was about 100–300 μm [28,29,30,31]. However, recently the pore size of 3D printing porous titanium is usually 500–1500 μm in clinical and basic research. Therefore, we proposed the concept of three-dimensional modification, which means constructing 3D micro-scaffolds in holes of the porous titanium scaffold. In this way, porous titanium and micro-scaffolds could provide mechanical support, optimal pore size and favorable microenvironment, respectively. Our results showed that new bone fulfilled the whole pores but not only on the inner surface, which was more obviously in the group with Gel:nHA = 1:1. All the results proved the feasibility and advantage of three-dimensional modification in the clinic.

As we all know, collagen and hydroxyapatite are the two most important components of bone matrix; gelatin is the denatured form of collagen that contains many functional amino acids and has almost identical composition as that of collagen [41,42]. Hydroxyapatite can enhance new bone formation by increasing osteoblast adhesion, proliferation, osteointegration, and calcium deposition [43,44]. As reported by Piergiorgio Gentile, nHA has higher surface area and surface roughness, resulting in better cell adhesion and cell–matrix interactions [26]. In the present study, three different proportion ratios of gelatin and nHA were used to construct hybrid scaffold, and all hybrid scaffolds showed significant promotion in cell adhesion, proliferation and differentiation. In particular, the group with Gel:nHA = 1:1 showed a multi-hierarchical pore structure with the average pore size of 156 ± 86 μm, which was most suitable for cell climbing and growth. This result was in consistent with Masaya Yamamoto′s finding [27,45]. In comparison, in the Gel:nHA = 1:0 group, because of the absence of hydroxyapatite, the pore was constructed by pure gelatin with a uniform size of 67 ± 32 μm. When contacting with liquid, porous gelatin swelled and pore size became more compact, which was not suitable for cell growth. As for the Gel:nHA = 1:3 group, the higher proportion of hydroxyapatite resulted in more collapsed areas and more invalid space in the scaffold. Above that, other explanations such as different forms of distribution and different topology of nHA may also influence cell response, which needs more in-depth study in the future.

It could be therefore concluded that Gel/nHA 3D micro-scaffolds may be a promising method for enhancing bone regeneration capability of porous titanium. It cannot only provide a suitable bone regeneration environment but also can be used as a carrier for bioactive factors such as VEGF to promote the performance of neovascularization. However, more attention should be paid for further optimization.

## 4. Materials and Methods

### 4.1. Porous Titanium Scaffolds

Porous titanium scaffolds were produced with Ti6Al4V powder (ASTM B348, grade 23) using selective laser melting (SLM; Concept Laser, Lichtenfels, Germany). Based on the CAD data, a dodecahedron was designed as the unit cell of the porous structures with a strut width of 300 μm and an average pore size of 1500 μm as well as a porosity of 84.8%. Four kinds of samples including disk-shaped samples (Ø5 mm and Ø30 mm, for *in vitro* assays), cylindrical samples (Ø5 mm × 10 mm for biomechanical tests) and flat cylinders (length 15 mm, for animal experiments) were produced, respectively. All samples underwent a post-production heat treatment. Properties of porous implant architecture (e.g., pore size, strut size and porosity) were verified by micro-CT (SkyScan 1076, Bruker Co, Kartuizersweg, Kontich, Belgium).

### 4.2. Incorporation of Gelatin/Nano-Hydroxyapatite (Gel/nHA) 3D Micro-Scaffolds into Porous Titanium

In our study, three groups of micro-scaffolds (3 proportion ratios of Gel/nHA) were prepared through chemical crosslinking with glutaraldehyde. Briefly, 3 wt. % gelatin (Type B, Sigma, St. Louis, MO, USA) and different concentrations of nHA (0, 3, and 9 wt. % of nHA; particle size = 20 nm, purity = 99%, Emperor Nanomaterial Co., Ltd., Nanjing, China) were mixed in deionized water and ultrasonically dispersed for 3 min at room temperature. After adding 0.625 wt. % of glutaraldehyde solution, porous titanium scaffolds were casted into solution and stirred quickly to force foam into each pore. Samples were kept at 4 °C for 12 h for gelatin crosslinking, then immersed in glycine solution at room temperature to block any residual glutaraldehyde, followed by washing 3 times with distilled water, freeze-drying at −80 °C and sterilization by ethylene oxide. Morphological characteristics were visualized using scanning electron microscopy (SEM; Nova NanoSEM 450, 5.00 kV).

### 4.3. In Vitro Cellular Assessments

#### 4.3.1. Cell Culture

The pre-osteoblast cells (MC3T3-E1) were purchased from Institute of Basic Medical Sciences, Peking Union Medical College (Chinese Academy of Medical Science, Beijing, China) and cultured in α-MEM medium (Gibco, Grand Island, NY, USA) supplemented with 10% fetal calf serum (FCS; Invitrogen, Carlsbad, CA, USA).

#### 4.3.2. Cell Attachment and Proliferation

Morphology of the adherent cells was observed by SEM. Briefly, cell suspension (2.5 × 10^5^ cells/mL, 40 μL) was dropped into the scaffold in a 96-well plate and cultured for 4 h. Then, medium was added and cells were incubated for 7 days with medium change every 2–3 days. Samples were rinsed with PBS buffer to remove non-adherent cells and then fixed with 2.5 wt. % glutaraldehyde for 1 h. A series of gradient ethanol solutions (50%, 70%, 90%, 95%, 100%) was used for sample dehydration and then replaced by pure isopentyl acetate. The morphology of cells were observed by SEM (FEI, Nova NanoSEM 450, 10.00 kV) and the samples were sputter coated with a 10 nm thick gold film before measurements.

Cell proliferation was measured by 4′,6-diamidino-2-phenylindole (DAPI; Sigma Co., St. Louis, MO, USA) fluorescent labeling and CCK-8 assay. At day 7, the whole scaffold was fixed and stained with DAPI for 15 min. DAPI fluorescent signals were viewed with a positive fluorescence microscope (Nikon Eclipse 80i, Inc., Tokyo, Japan). The cell area and cell density were calculated with six different region of interest (ROI) fields of each sample (*n* = 3) from immunofluorescence imaging using ImageJ software (NIH, Bethesda, MD, USA). To quantify cell proliferation levels, cells were assessed by using CCK8 assay Kit (Dojindo Molecular Technologies Inc., Minato-ku, Tokyo, Japan) after cell culture for 1, 3 and 7 days.

#### 4.3.3. Cell Differentiation

Osteogenesis differentiation of attached cells were assessed by RT-PCR and ELISA at gene and protein levels, respectively. On disc samples, the cells were cultured for 14 days and harvested (*n* = 3). Total RNA was extracted using TRIZOL reagent (Invitrogen) and reversed into cDNA using the cDNA synthesis kit (Thermo Scientific, Waltham, MA, USA). Expression levels of osteoblastic markers (alkaline phosphatase (ALP), collagen type 1 (Col-1), osteocalcin (OC)) and angiogenic marker (vascular endothelial growth factor (VEGF)) were quantified using SYBR green master mix (KAPA Biosystems, Wilmington, MA, USA) and a StepOne Plus RT-PCR instrument (ABI, Carlsbad, CA, USA). Expression levels were calculated based on the 2^−ΔΔ*C*t^ method by normalizing values to the housekeeping gene, β-actin. Primers used for the selected genes were shown as follows:

ALP (F:5′-GGCAACTCCATCTTTGGTCTG-3′; R:5′-GCCTGGTAGTTGTTGTGAGCGT-3′); Col-1 (F:5′-ATGACCGATGGATTCCCGTTC-3′; R:5′-ACGCTGTTCTTGCAGTGATAGGT-3′); (F:5′-CGGGAGCAGTGTGAGCTTAAC-3′; R:5′-CAAAGCCGAGCTGCCAGAGT-3′); VEGF (F:5′-TCTGTGTTTCCAATCTCTCTCTCC-3′; R:5′-CTTATTTCAAAGGAATGTGTGGTG-3′).

Commercial ELISA kits (ALP, abcam, Cambridge, UK; Col-1, Cloud-Clone Corp, Houston, TX, USA; OC, LifeSpan BioSciences, Seattle, WA, USA; VEGF, abcam, Cambridge, UK) were used to further verify the different osteogenic properties among four groups. The medium supernatants after 14 days of culture (*n* = 3) were processed according to the kit instructions, and results were calculated from a standard absorbance curve at 450 nm.

### 4.4. In Vivo Assessment of New Bone Formation

#### 4.4.1. Implantation Procedure

All animal experiments were approved by the animal care committee of Peking Union Medical College Hospital. Twenty-four healthy skeletally mature female New Zealand White rabbits (aged 20 weeks; weight range: 3.2–3.6 kg) were randomly divided into four groups (6/group), and received porous titanium implants filled with (1) nothing (control); (2) micro-scaffolds with Gel:nHA = 1:0; (3) micro-scaffolds with Gel:nHA = 1:1; and (4) micro-scaffolds with Gel:nHA = 1:3. The rabbits were anesthetized with 10% chloral hydrate (1.2 mL/kg intravenously). A 15 mm critical-size segmental defect was made in the radius with a surgical line saw supplemented by copious 0.9% sterilized saline irrigation. The surrounding periosteum was removed over radial segment completely, and scaffolds were implanted press-fit into the defect without additional fixation devices. The incisions were closed in layers and dressed with bandages.

#### 4.4.2. Micro-CT Evaluation

Bone regeneration was measured by *ex vivo* micro-CT scans (SkyScan 1076; Bruker micro-CT N.V., Kontich, Belgium) on isolated grafted radius at 12 weeks postoperatively. Individual bony blocks containing the implants and the surrounding tissues were obtained after euthanizing rabbits, and fixed in 4% formaldehyde solution for two weeks. *Ex vivo* micro-CT images were acquired at 30 μm resolution (voltage: 70 kV, current: 141 μA, 1.0 mm Al filter, rotation step: 0.6) and then reconstructed using volumetric reconstruction software NRecon version 1.6.6 (Bruker micro-CT N.V., Kontich, Belgium). Bone regeneration was expressed as bone volume (BV), total volume (TV) and percent bone volume (BV/TV) using CTAn software (ver 1.13, Bruker micro-CT NV, Kontich, Belgium).

#### 4.4.3. Histological Processing and Histomorphometry

After micro-CT evaluation, histological analysis was performed on all specimens to investigate the interface of bone and titanium and bone morphology. Samples were dehydrated in a graded ethanol solution from 70% to 100%, and finally embedded in methyl methacrylate. Sections with thickness of 50 μm were obtained using a modified interlocked diamond saw (Leica Microtome, Wetzlar, Germany) and stained with 1.2% trinitrophenol solution as well as 1% acid fuchsin solution (Von-Gieson staining).

### 4.5. Statistical Analysis

SPSS Statistics 21.0 (SPSS, Inc., Al Monk, NY, USA) was used for statistical analysis. Data were presented as means ± standard deviations. One-way ANOVA and subsequent *post hoc* Tukey′s test was selected to investigate differences among four groups. Statistical significance was declared as (*) at *p* < 0.05, (**) at *p* < 0.01, (***) at *p* < 0.001, (****) at *p* < 0.0001.

## 5. Conclusions

This study demonstrated an innovative Gel/nHA 3D micro-scaffold modification method for porous titanium. All hybrid scaffolds with different composition ratios were found to significantly enhance cell adhesion, proliferation and differentiation as well as the regenerated bone volume *in vivo*. Gel:nHA = 1:1 group showed the best performance. All of these findings indicated that Gel/nHA 3D micro-scaffold modification hybrid scaffold had a good biocompatibility and bone regeneration capability, which may potentially be applied in the clinic.
